# Supplementary material for: Prevalence of Wheezing and Its Association with Environmental Tobacco Smoke Exposure among Rural and Urban Preschool Children in Mpumalanga Province, South Africa
Source: Int J Environ Res Public Health. 2024 Apr 11;21(4):469. doi: 10.3390/ijerph21040469 (PMC11050571; doi:10.3390/ijerph21040469)
Supplement: Supplementary file 1 [file ijerph-21-00469-s001.zip › ijerph-2913967-supplementary.pdf]

Supplementary Materials

Table S1: Total number of pre-schoolers per district (Pre-Covid-19 and data collection)

Municipality name	Area type	Number of pre-schoolers per area type	Total number
Chief Albert Luthuli	Urban<br>Rural	360<br>770	1 130
Dipaleseng	Urban<br>Rural	283<br>991	1 274
Govan Mbeki	Rural	5451	5 451
Lekwa	Rural	1 516	1 516
Mkhondo	Urban<br>Rural	443<br>1 418	1 861
Msukukaligwa	Rural<br>Urban	90<br>1 380	1 470
DR Pixley ka Seme	Urban<br>Rural	157<br>626	783
			13 485

Table S2: Sample size calculation (Pre-Covid-19 and data collection)

Municipalities	Number of Rural pre-schoolers	Percentage contribution of municipality to the total population%	Required Sample number(n)
Chief Albert Luthuli	770	5,7%	223
Dipaleseng	991	7,3%	287
Govan Mbeki	5451	40,4%	1576
Lekwa	1516	11,2%	438
Mkhondo	1418	10,5%	410
Msukukaligwa	1380	10,2%	399
DR Pixley ka Seme	626	4,6%	181
Total	***12 152***	***90,1%***	***3 514***
**Municipalities**	**Number of Urban pre-schoolers**	**Percentage contribution of municipality to the total population%**	**Required Sample number(n)**
Chief Albert Luthuli	360	2,67%	105
Dipaleseng	283	2,10%	82
Govan Mbeki		0,00%	0
Lekwa		0,00%	0
Mkhondo	443	3,29%	128
Msukukaligwa	90	0,67%	26
DR Pixley ka Seme	157	1,16%	45
Total	***1 333***	***9,89%***	***386***
**Grand Total**	**13 485**		**3 900**

Table S3: Data collection using the calculated sample size.

Municipalities	Number of Rural pre-schoolers (actual number)	Percentage contribution of municipality to the total population%) (pre-covid 19)	Required Sample number(n) (pre-covid 19)	Response number
Chief Albert Luthuli	554	5,7%	223	173
Dipaleseng	764	7,3%	287	200
Govan Mbeki	3567	40,4%	1576	960
Lekwa	1022	11,2%	438	297
Mkhondo	927	10,5%	410	340
Msukukaligwa	934	10,2%	399	301
DR Pixley ka Seme	346	4,6%	181	101
Total	***8 114***	***90,1%***	***3 514***	***2 372***
**Municipalities**	**Number of Urban pre-schoolers (actual number)**	**Percentage contribution of municipality to the total population%) (pre-covid 19)**	**Required Sample number(n) (pre-covid 19)**	**Response number**
Chief Albert Luthuli	315	2.67%	105	265
Dipaleseng	219	2.10%	82	163
Govan Mbeki		0,00%		
Lekwa		0.00%		
Mkhondo	334	3,29%	128	232
Msukukaligwa	72	0,67%	26	32
DR Pixley ka Seme	126	1,16%	45	81
Total	***1 066***	***9,89%***	***386***	***773***
**Grand Total**	**9 180**		**3 900**	
